# Supplementary material for: Asymmetric Synthesis of Quaternary Hydantoins via a Palladium-Catalyzed Aza-Heck Cyclization
Source: J Am Chem Soc. 2025 Nov 14;147(49):44692–8. doi: 10.1021/jacs.5c16022 (PMC12703750; doi:10.1021/jacs.5c16022)
Supplement: Supplementary file 2 [file ja5c16022_si_002.zip › All NMR FID Files/S33/S33_CarbonNMR/1/pdata/1/email_EM05-089-13C-DMSO_1_1.pdf]

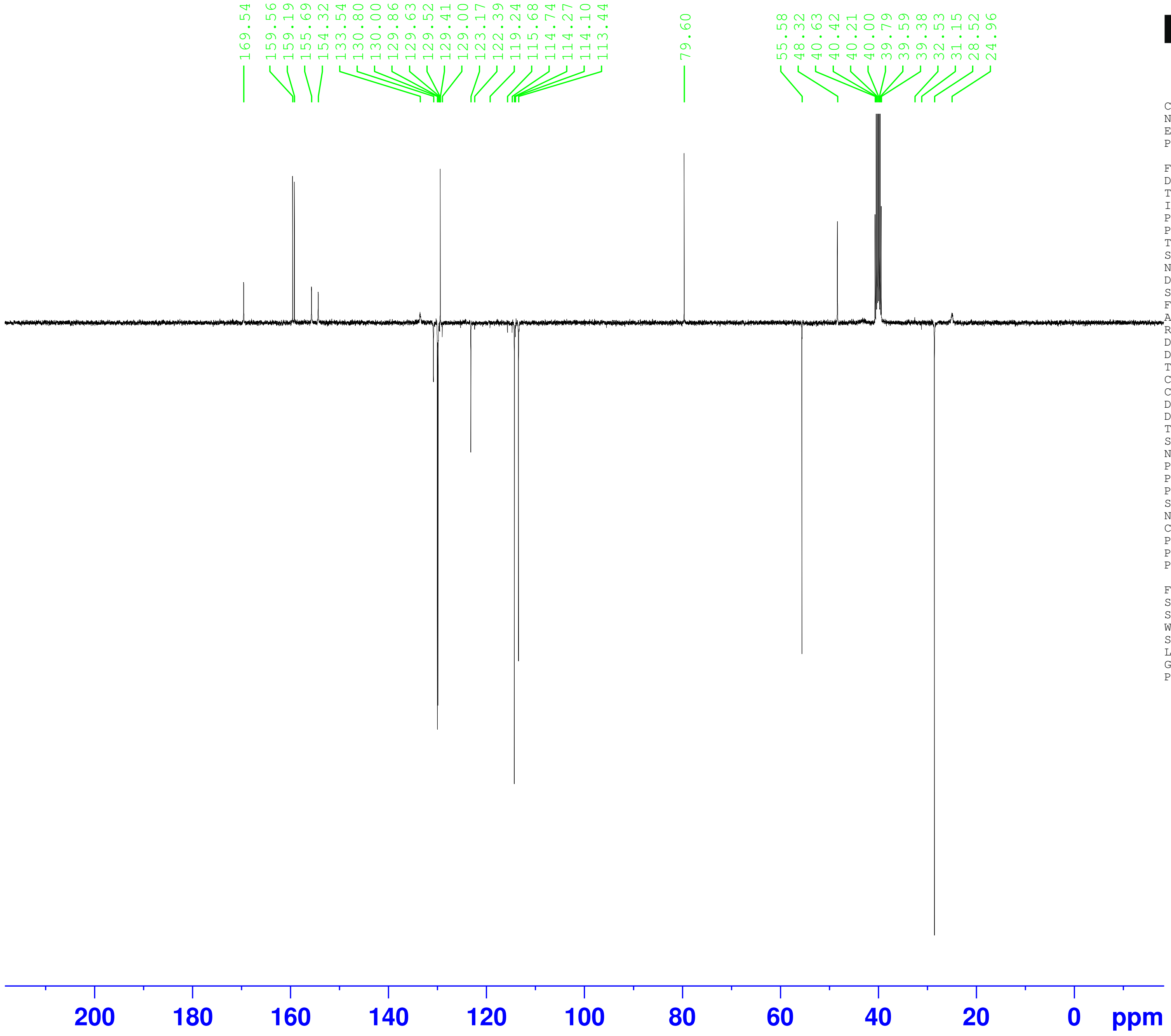


```
Current Data Parameters
NAME       EM05-089-13C-DMSO
EXPNO                     1
PROCNO                    1

F2 - Acquisition Parameters
Date_              20240321
Time                   7.23 h
INSTRUM   Avance Neo 400 U of DE Brown Lab
PROBHD    Z104450_0412 (
PULPROG                jmod
TD                    65536
SOLVENT                DMSO
NS                     2048
DS                        4
SWH               23809.524 Hz
FIDRES             0.726609 Hz
AQ                1.3762560 sec
RG                      101
DW                   21.000 usec
DE                     6.50 usec
TE                    298.0 K
CNST2           145.0000000
CNST11            1.0000000
D1               5.00000000 sec
D20              0.00689655 sec
TD0                       1
SFO1            100.6228298 MHz
NUC1                    13C
P1                    10.00 usec
P2                    20.00 usec
PLW1            44.93500137 W
SFO2            400.1316005 MHz
NUC2                     1H
CPDPRG[2            waltz65
PCPD2                 90.00 usec
PLW2             8.31639957 W
PLW12            0.23101000 W

F2 - Processing parameters
SI                    32768
SF              100.6127685 MHz
WDW                      EM
SSB                       0
LB                     1.00 Hz
GB                        0
PC                     1.40
```
